# Probe-level linear model fitting and mixture modeling results in high accuracy detection of differential gene expression

**DOI:** 10.1186/1471-2105-7-391

**Published:** 2006-08-25

**Authors:** Sébastien Lemieux

**Affiliations:** 1 Institute for Research in Immunology and Cancer, Université de Montréal, Montréal, Canada

## Abstract

**Background:**

The identification of differentially expressed genes (DEGs) from Affymetrix GeneChips arrays is currently done by first computing expression levels from the low-level probe intensities, then deriving significance by comparing these expression levels between conditions. The proposed PL-LM (Probe-Level Linear Model) method implements a linear model applied on the probe-level data to directly estimate the treatment effect. A finite mixture of Gaussian components is then used to identify DEGs using the coefficients estimated by the linear model. This approach can readily be applied to experimental design with or without replication.

**Results:**

On a wholly defined dataset, the PL-LM method was able to identify 75% of the differentially expressed genes within 10% of false positives. This accuracy was achieved both using the three replicates per conditions available in the dataset and using only one replicate per condition.

**Conclusion:**

The method achieves, on this dataset, a higher accuracy than the best set of tools identified by the authors of the dataset, and does so using only one replicate per condition.

## Background

DNA microarrays are commonly used to measure, in parallel, the steady-state concentration of tens of thousands of mRNAs, providing an estimate of the level of expression of the corresponding genes. They come in two flavors: 1) spotted arrays allows for the simultaneous measurement of two samples on the same array, we'll refer to these arrays as multi-channel arrays; 2) Affymetrix GeneChips arrays with a significantly higher density but only allowing for the hybridization of one sample, we'll refer to those as High-Density Arrays, HDAs. A typical piece of information that investigators seek to extract from microarrays is the list of differentially expressed genes (DEGs) between a treatment and a control condition. This can either take the form of a defined subset or an ordering of the whole transcriptome on which some meaningful (statistically and/or practically) threshold is applied. Until recently most of the efforts to derive a statistical method to solve this problem have been focusing on a general approach applicable to both type of arrays. The strategy when analyzing HDAs is to estimate the absolute level expression of a given gene in each condition and then compute the log-ratio of the expression levels between two conditions. Methods applied to log-ratios on multi-channel arrays can readily be applied to these computed ratios.

A fundamental issue when analyzing microarrays data is the imbalance between the dimensionality of the data (tens of thousands of measures for each sample) vs. the number of samples or replicates (for most studies between 2 and 60). On the theoretical side, this imbalance gives rise to the necessity of adjusting statistical thresholds to the context of multiple testing [1,2], seriously complicating the accurate estimation of sensitivity and specificity. Because widely used statistical methods are based on an estimate of the gene-specific variation, they technically require at least 2 replicates per conditions. In the context of multi-channel arrays, experts in the field have been recommending the use of 6 replicates [3]. But, for practical reasons, most laboratories have been settling for 3 replicates per condition. Proof-of-principle experiments with no replication (one array per condition) are still performed routinely by several labs and typically analyzed by the simple method of identifying genes as putative DEGs if they show a ratio above 2 or below 0.5.

In the work presented here, a significant increase in the accuracy of DEGs detection from a low number of replicate arrays will be obtained by taking advantage of the design itself of HDAs. All of the Affymetrix GeneChips contain *k *pairs of 25-mer DNA oligonucleotides, called probe pairs, per probe-set. The probe-sets are designed to measure the expression of a single gene. Each probe pair is composed of a match (PM) and mismatch (MM) probe, the former being the exact 25 nucleotides and the later containing one single mismatch at position 13. MM probes were introduced to estimate the level of non-specific hybridization of the corresponding PM probe, but the recent trend has been to ignore the MM measures and only use PM measures seeking alternative methods to estimate non-specific hybridization. The measure of interest being the level of expression of the genes, it is necessary to transform a set of *k *probe pair's intensities into a single probe-set measurement. This step is referred to as the expression summary. It is still debated which expression summary method gives the best results but both MAS5.0 [4] and RMA [5] have been widely used. A recent summarization approach, FARMS [6], have been found to compare favorably to more classical summarization approaches and will be included in the comparisons.

The publication of the Choe *et al*. dataset in 2005 [7] has provided an objective benchmark to evaluate the accuracy of differential gene expression of various combinations of methods. In this work the authors have amplified 3,866 cRNAs and prepared two samples, 'control' (C) and 'spike' (S), where 1,331 cRNAs were spiked at various ratios of concentrations from 1.2 to 4. The two samples were then hybridized to three Affymetrix GeneChips and scanned using standard Affymetrix protocol. The work was done using the Affymetrix *Drosophila *array (DrosGenome1) where each gene is represented by a probe-set of 14 probe pairs of 25-mer DNA oligonuceotides. There are a total of 14,010 probe-sets present on the array. Two key characteristics of this dataset will be important to remember when carrying out the statistical analysis: 1) a large proportion (72%) of the probe-sets corresponds to cRNAs that were not present in the samples, leading to a bimodal intensity distribution; 2) Only concentration ratios above 1 were introduced in the spiked sample, inducing a strong asymmetry in the distribution of log-ratios. Using this validation dataset, it was shown [7] that a preferred set of steps can be identified to optimize the detection of DEGs in HDAs. The optimal pipeline identified corresponds to the use of MAS5.0 for the background correction, probe-level normalization, PM adjustment and the use of the medianpolish algorithm for expression summaries (borrowed from RMA[5]), followed by a loess normalization on the probe-set level data and finally use Cyber-T [8] to identify DEGs.

The most successful and widely used methods to identify DEGs are currently based on variations of a regularized *t*-statistic where the standard deviation term is weighted [8], a constant added to it [9,10] or both [11]. Regularization terms are always, but in various ways, estimated from the dataset being analyzed to account for experiment-specific behavior of the data. Recent work from Barrera *et al*. [12] attempts to identify DEGs directly from the probe-level data using a two-way ANOVA approach. Their results suggest that this class of approaches can outperform probe-set level methods or give comparable results with less replicates, but their validation relies on the use of the Affymetrix Latin Square dataset which contains DEGs with concentration ratios of 2 and above on a small number of genes (a total of 126 spiked genes). All statistical methods tested (data not shown) perform well on this dataset and it is often difficult to quantify slight improvements in accuracy.

In this paper, I propose a method that scores better accuracy than the preferred pipeline identified by Choe *et al*. [7] and will demonstrate that this method doesn't require the three technical replicates provided by the dataset to correctly identify 75% of the DEGs within 10% of false positives. The proposed method, called PL-LM (Probe-Level Linear Model), directly models the treatment effect versus a baseline control using the probe-level data, using a linear model. The treatment effect and average intensity fitted by the linear model are then modeled by a Gaussian Mixture Model (GMM), which is used to separate the DEGs from non-DEGs. The outcome of the GMM is the probability of each probe-set to belong to the cluster of DEGs, thus the PL-LM method as a whole should be characterized as a feature selection algorithm. This second stage of the analysis borrows idea introduced by Jia and Xu [13], where they used a GMM to cluster genes showing similar expression responses vs. a quantitative phenotype. The average intensity and treatment effect in PL-LM are equivalent to the β_k0 _and β_k1 _in [13]. There are a few fundamental differences between the two approaches: 1) in PL-LM, the optimization of the GMM is decoupled from the fitting of the linear model, 2) the linear model of PL-LM is based on probe-level data, and 3) the clustering step aims essentially at separating DEGs from non-DEGs.

## Results and discussion

**Figure 1:**
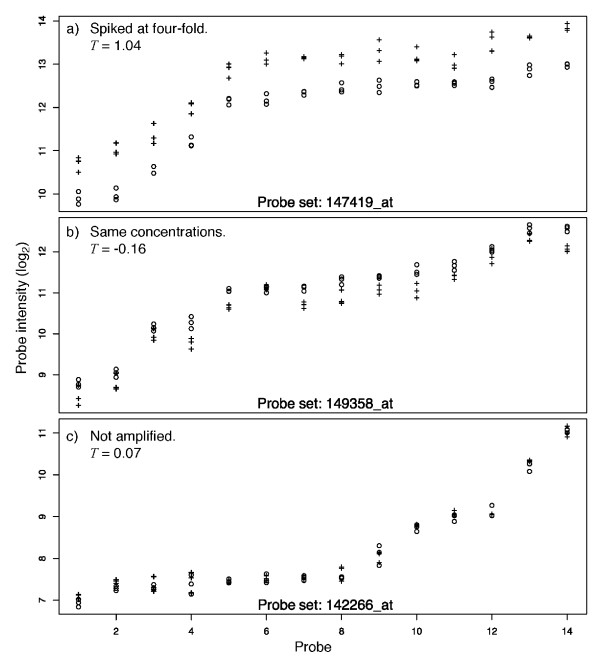
Examples of probe-level data. a) For a differentially expressed gene (cRNA spiked at 4-fold from the Choe *et al*. dataset, probe-set: 147419_at), b) a non-differentially expressed gene (cRNA at equal concentrations, probe-set: 149358_at), and c) a non-expressed gene (not in the pool of amplified cRNAs, probe-set: 142266_at). Quantile-normalized data from the control (circle) and spiked (+) samples are shown, including replicate data. Probes are ordered by the average intensity on the control replicates. For each probe-set, the value of *T *obtained from the linear model is shown.

Data from the Choe *et al*. [7] dataset was log_2_-transformed, quantile-normalized [14] and a linear model (see Methods section) was fit for each probe-set, estimating parameter *T*, the treatment effect in equation 2 using a standard least-square method [15]. Figure 1 presents normalized probe-level data for three typical probe-sets: a) a probe-set that was spiked at four times the concentration of the control; b) a probe-set that wasn't spiked, and c) a probe-set corresponding to a cRNA that wasn't amplified. For each probe-set the value estimated for the treatment effect, *T*, is reported. A first observation that can be made is the fact that values obtained for *T *underestimate the ratio of concentration (*T *= 1 would be equivalent to a two-fold ratio). This trend has been also reported by Choe *et al*. [7] for other methods on their dataset. It seems to be either a limitation introduced by the impossibility to accurately estimate non-specific signal for each probe or a real bias introduced by the experimental methods to prepare the dataset. Another important observation is that cRNAs that were not spiked tend to have a negative value of *T*, suggesting that they are seen as decreasing in the spiked vs. the control sample. Examination of the raw data presented in figure 1b confirms this trend since for each probe, all three spiked replicates fall below the intensities of the three control replicates. On the other hand, for probe-set corresponding to cRNAs that were not amplified the intensities seem to be similar between the spiked and control samples (figure 1c).

**Figure 2:**
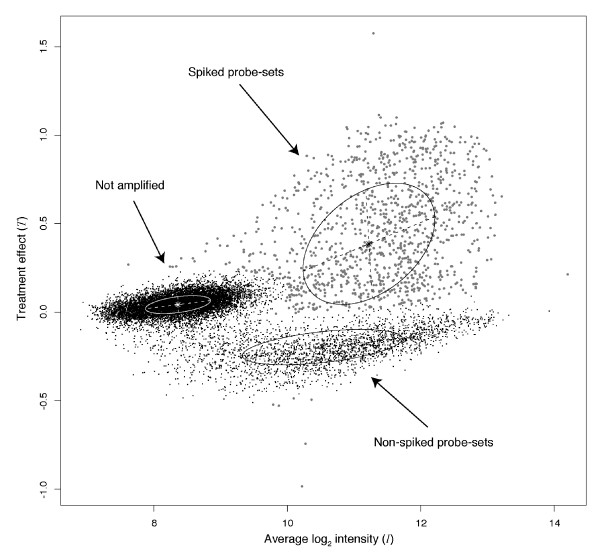
Mixture modeling of the PL-LM method on the Choe *et al*. dataset. The three mixture components are represented as ellipse identifying their center and variances. Data points with a conditional probability of belonging to the component modeling DEGs above 0.5 are shown as larger gray dots. The mixing proportions of the three components are 17%, 74% and 9%, respectively for the non-spiked probe-sets, not amplified cRNAs and DEGs.

For each probe-set, the average log_2 _intensity is also determined by considering all probes and will be referred as *I*. To identify DEGs, a mixture model with three Gaussian components (with variable mean, and full variance matrices) was fitted to the two-dimensional data points defined by *T *and *I*. The number of Gaussian components to use in the mixture is dictate by the distribution of data points with respect to *T *and *I *as observed from a scatter plot. Figure 2 shows the result of this model. Upon multiple trials, starting from random assignations of data points to mixture components, the same classification was obtained (data not shown). The three mixture components are represented in figure 2 as ellipses, visually translating the mixtures parameters. Not shown in the figure, the mixing proportions of the three components are 17%, 74% and 9%, and can be respectively attributed to non-spiked probe-sets, not amplified cRNAs and DEGs. Probe-sets having a conditional probability of belonging to the third component above 0.5 are identified by larger dots and grayed out. This conditional probability will be used as the ordering statistic to compute ROC curves and allows for an objective comparison with other methods to identify DEGs. Observations made on the three typical probe-sets of figure 1 can be seen as widespread among their classes. There also seem to be a slight intensity-dependant bias over *T *that can be observed in all three components.

**Figure 3:**
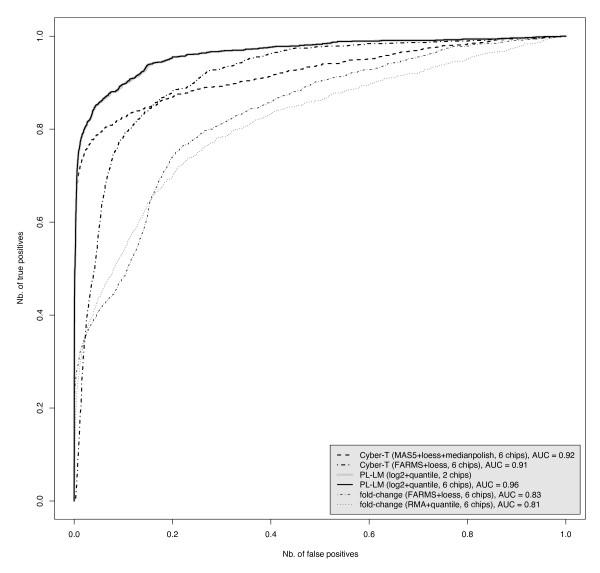
ROC curves comparing methods on the Choe *et al*. dataset. Probe-sets with a spiked to control concentration ratio above or equal to 1.2 were considered as DEGs to compute these curves, resulting in 1,326 spiked probe-sets to identify among a total of 14,010. The fold-change applied after RMA summaries and quantile normalization is shown as a baseline for comparison since it is a simple and frequently used method. Nine shaded curves, representing the results from applying the PL-LM method to all combinations of two arrays (one control vs. one spike), are falling directly under the PL-LM (on all replicates) curve (black line). For all methods, the area under the curve (AUC) is reported as a quantitative measure of both sensitivity and specificity.

To assess the accuracy of the ordering induced by the PL-LM method, ROC curves were computed for results obtained by using all six arrays from the dataset and for all combinations of two arrays (one control and one spiked sample). As a comparison, three other approaches were applied to the dataset: 1) the preferred pipeline identified by Choe *et al*. [7] using the MAS5.0 algorithm [4] but using the medianpolish as the summarization step, loess normalization [16] and Cyber-T regularized *t*-statistic [8]; 2) a simple approach combining the RMA expression summary [5], quantile normalization [14] and absolute fold-change ordering; 3) the FARMS summarization method, with the ordering obtained from either the absolute fold-change or by applying the cyber-T approach. Resulting ROC curves are shown in figure 3. In an attempt to quantify the overall accuracy of each method, the area under the curve (AUC) of the ROC curves have been computed and reported in the legend. First, both the PL-LM (on two or six arrays) and Cyber-T perform equally well up to the identification of around 800 true positives, after which the PL-LM significantly outperforms the Cyber-T method whether using all arrays or a single replicate per condition. The fold-change method applied to quantile-normalized RMA summaries, even when using three replicates per condition, performs on a much lower level than Cyber-T, highlighting a clear necessity for a proper statistical analysis when reporting HDA data, even in the absence of replication.

**Figure 4:**
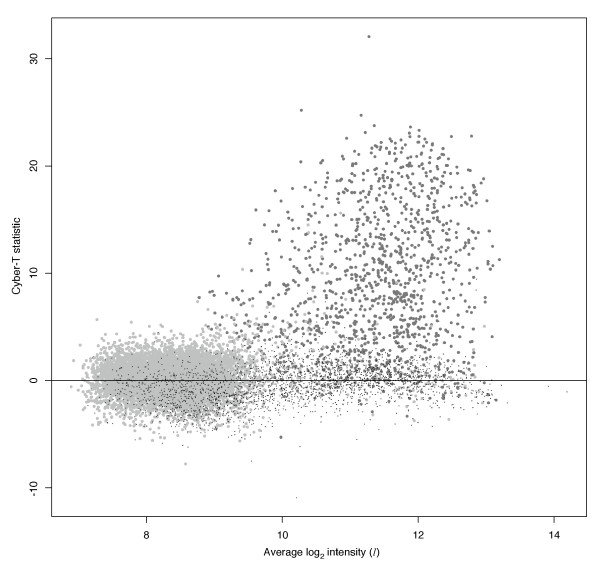
Distribution of the Cyber-T statistic as a function of average intensity *I*. Spiked probe-sets are shown as large and dark gray dots, not amplified cRNAs as large and light gray dots, and probe-sets that where not spiked as small black dots.

To assess if a higher level of analysis on the Cyber-T regularized *t*-statistic could help distinguish between DEGs and non-DEGs, the distribution of this statistic vs. the average intensity *I *is presented in figure 4. Spiked probe-sets are shown as large and dark dots, not amplified cRNAs as large and light dots, and probe-sets that where not spiked as small black dots. An interesting feature of Cyber-T is that it was able to completely remove the intensity-dependant bias on its statistic. But, in doing so, seems to blur the boundaries between the clusters identified from the PL-LM method. It is also important to notice that the clear clustering apparent in figure 2 is revealed only by the use of the treatment coefficient *T *and is mostly lost using probe-set-based statistics.

## Conclusion

By adapting and combining previously proposed approaches (probe-level linear model [12] and treatment effect clustering [13]), the PL-LM method was able to outperform by a significant margin the preferred method identified by Choe *et al*. [7] on their validation dataset: MAS5.0 background correction and PM adjustment, median polish expression summaries followed by loess normalization and Cyber-T [8]. Moreover, by using probe-level data to assess the variability of a differential expression measure, the PL-LM method maintains its level of performance on the validation dataset even when only one array per condition (control and spike) is used, no matter which arrays are chosen. These results indicate that, within the experimental setup used for the validation study of Choe *et al*., no replication was necessary to correctly identify 75% of the DEGs within 10% of false positives (compared to 69% using Cyber-T and 14% using the fold-change method).

It is important to qualify the previous statement since the replicates that were performed in this study were technical replicates obtained by hybridizing the same pool of labeled cRNAs to three arrays: the PL-LM method doesn't require technical replicates to achieve high accuracy. In several contexts, it will still be recommended to perform biological replicates to account for possible variations due to limitations of the experimental protocol (small amount of material available, uncontrolled biological factors, etc.). But in general, if each biological sample provides a statistically sound sampling of the population to study, this variability should be negligible. Despite being a controversial technique [17-20], it is possible in practice to reduce this variability by increasing the amount of RNA per sample, and can readily be achieved by pooling multiple samples together. The downside of this approach is that quantification of the biological variations not due to the factor of interest is lost, but this loss might be more than compensated by the reduction in array costs. In fact, to properly quantify the biological variation and to avoid relying on *a priori *assumptions to regularize it, typically requires a number of arrays that falls well above the budget of a large number of studies. In the study on pooling carried out by Jolly *et al*. [17] they compare an ANOVA analysis performed on probe-set expression levels obtained from 5 vs. 3 samples with the analysis of pooled RNA done based on the observed fold-change. They concluded that the pooled strategy should only be used "where the expected response or phenotype is robust and its variation in that response is minimal". It is debatable whether their conclusions stems from the fundamentally diverging approaches used to analyze the pooled vs. individuals datasets or if it truly reflects limitations set forth by the pooling process. The PL-LM method, by being amenable to both multiple and single replicate context, could be used to remove the method bias when comparing the pooled vs. non-pooled approach.

The PL-LM method still carries a few drawbacks. The most limiting aspect is the manual nature of the mixture modeling step: deciding on the number of components and which component(s) is (are) modeling the DEGs is currently done by manual inspection of the optimized mixture parameters. In a different context, Jia *et al*. [13] have been using the Bayesian information criterion (BIC) to determine the optimal number of components. The use of a graphical representation as shown in figure 2 greatly simplifies this step but, depending on the dataset analyzed the group separation might be less obvious, and mostly, the procedure relies on the knowledge of it operator. It is also a possibility that on a dataset with an extremely low proportion of DEGs (1 to 10 for example), the mixture step will require a large number of components before dedicating one to the DEGs, complicating the visual assessment of the mixture model. In such circumstance, the mixture could be used to model non-DEGs and an outlier detection approach used to identify DEGs (see [21]).

In essence, the PL-LM method implements in a single step the RMA expression summary [5] and the linear model proposed by Kerr *et al*. [22]. By quantifying directly the difference induced by the treatment without the need to estimate the expression level of a probe-set for each array, the method achieves, on a wholly defined validation dataset, better accuracy without any technical replicate. The reduction in the number of estimated parameters and possibility to consider across treatments observations increases the robustness of the approach to quantify the treatment difference.

## Methods

### Normalization

Two normalization methods were tested as a pre-treatment to PL-LM to remove intensity-dependant bias and scaling effect: 1) Cyclic loess, introduced by Dudoit *et al*. [1], qualitatively performed the best on the Choe *et al*. dataset but requires 5 minutes of computation time (5 cycles were applied, all CPU time reported are from a P4 3.2GHz); 2) Quantile normalization, described in Bolstad *et al*. [14], requires 10 seconds of computation time and returns quantitatively equivalent results after applying the PL-LM and mixture model. Unless stated, all results presented in this paper were done on a log_2_-transformed and quantile-normalized dataset.

### Overview of PL-LM

The process of segmenting the genes represented on an array between DEGs and non-DEGs is a highly debated subject. Feature selection can actually be broken down into two distinct steps, a first step seeks to order the genes from the most susceptible to be a DEG to the least, most often returning a transformation of the primary data into a single scalar (called the ordering statistics). As examples, the log_2 _fold-change, *t*-statistics, z-scores, relative differences and *p*-values used by various approaches all play this role. Then, an appropriate threshold should be determined to separate the DEGs from the non-DEGs. Typical approaches are the *ad hoc *choice of a threshold (the two-fold change rule), *ad hoc *choice of a number of genes to consider as DEGs, choice of an upper bound to the false discovery rate [2], or to the Bonferroni-adjusted *p*-value. Contrarily to common belief, the computation of the ordering statistics doesn't need to be quantitatively accurate, but only its relation to the determined threshold is of importance. The main feature sought in an ordering statistic is its power to segregate the DEGs from non-DEGs. In this paper the discussion will be strictly limited to a choice of statistics since in most practical settings the resources available for follow-up experiments is the determinant factor in adjusting the threshold. When the true segmentation between DEGs and non-DEGs is known (as is the case with the Choe *et al*. dataset), the use of receiver-operator characteristic (ROC) curves provides visual assessment of the trade-offs between sensitivity and specificity of the ordering induced by a statistics without the need for a specific threshold. The area under the ROC curves (AUC) have also been computed for the approaches applied, it provides a quantitative evaluation of the performance of the method.

Linear model fitting has already been explored for the identification of DEGs in DNA microarray data [12,22,23]. It represents a flexible framework where a linear equation can be defined as a function of hidden parameters to return the observed data. Given enough data, well known methods [15] exist that are able to estimate values for each parameter that minimize the discrepancies between predicted output of the function and the observed data. When analyzing microarray data, the model is designed in such a way that one (or several) of the hidden parameters represents the treatment effect. Either this estimated parameter or the *p*-value returned by performing hypothesis testing on its value are then used as the ordering statistic.

The machine learning literature [24] has always been concerned with the concept of over-fitting, which is also applicable to linear model fitting. This concept states that using a too simple model (with less hidden parameters) leads to a systematic bias in the predictions since parts of the phenomenon to model are not accounted for. On the other hand, using a too complex model can lead to instability in the predictions, values of the hidden parameters are artificially adjusted to fit observed variations that are actually due to a normal noise. This situation is called over-fitting and typically arises when the number of observations becomes small with respect to the number of hidden parameters to estimate. The main impact of over-fitting in the context of linear models is a loss of accuracy in the hidden parameter estimates, which implies local shuffling on the order induced by the estimated statistic. An appropriate method to control over-fitting is to settle for a simpler model when the data is scarce, achieving a favorable trade-off between the bias introduced by the simpler model and the variability of the more complex model.

The PL-LM method uses an extra step on top of the linear model, finite mixture model [21] are used to transform the output of the linear model into an ordering statistics that can flexibly account for biases introduced by the experimental design. Since it is a well-known fact that a finite mixture of Gaussian components can model any arbitrary distribution, this approach doesn't make any assumption with respect to distributional properties of the data analyzed. The EM algorithm [25] implementation used in this work is the MCLUST software [26] running under R.

### Linear model

The first stage of the PL-LM approach implements a linear model sharing some level of resemblance to the two-way ANOVA implemented by Barrera *et al*. [12] on probe-level data. Their nomenclature will be used to avoid confusion and simplify the identification of differences between the approaches. In the Barrera *et al*. model, the log_2 _intensity observation for probe *i *of a given probe-set in array *k *corresponding to treatment *j *is modeled by the following linear equation:

*Y*_*ijk *_= μ + *P*_*i *_+ *T*_*j *_+ *PT*_*ij *_+ ε_*ijk*_,     (1)

where μ is the level of expression of the gene corresponding to this probe-set, *P*_*i *_models the effect of each probe affinity, *T*_*j *_represents the impact of the treatment *j*, *PT*_*ij *_is the specific effect of the treatment *j *on probe *i *and ε_*ijk *_is assumed to be normally distributed with a probe-set specific variance. In context of the Choe *et al*. dataset (2 treatments, triplicates each), fitting the model for a probe-set means estimating 1 + 14 + 2 + 28 = 40 parameters from 14 × 6 = 84 data points. In their work, Barrera *et al*. [12] suggest that decision should be taken by testing the hypothesis of equality between the treatments.

The PL-LM method seeks to first simplify the model to reduce the possibility of over-fitting the data. To identify DEGs, only the difference between a treatment and a control needs to be estimated. The equation is replaced by the following simplified version:

*Y*_*ijk *_= α_*i *_+ *T*_*j *_+ ε_*ijk*_.     (2)

Here, α_*i *_combines both the expression level of the gene and specific probe affinities, *T*_*j *_represents the effect of treatment *j*. *T*_*j *_will be constrained to 0 for the control treatment resulting in a simpler interpretation of *T*_*j *_for the other treatments, and making the solution to the least-square formulation unique. The error term ε_*ijk *_will absorb any probe-treatment specific effect and is assumed to be normally distributed with a probe-set specific variance. With this model a total of 14 + 1 = 15 parameters will be fitted, using standard least-square procedure, from the same 84 data points. In the context of a single treatment vs. control, the least-square estimators can be obtained analytically:

*T*_*j *_= *avg*_*ik *_(*Y*_*ijk *_- α_*i*_)

= *avg*_*ik *_(*Y*_*ijk*_) - *avg*_*i *_(α_*i*_)

α_*i *_= *avg*_*jk *_(*Y*_*ijk *_- *T*_*j*_)

= *avg*_*jk *_(*Y*_*ijk*_) - *avg*_*j *_(*T*_*j*_)

In the context of microarray data analysis, the appropriateness of the normally distributed error term, and thus the possibility to use least-square fitting procedure, is still under debate. The use of a mixture modeling step (see below) in PL-LM to derive the final ordering of the probe-set allows to account for biases introduced in the fitting of *T*, if this bias is shared by all probe-sets or if it is probe-set intensity-dependant.

This model can readily be extended to experimental designs testing multiple treatments (having more than one unconstrained *T*_*j*_) and can be applied in the absence of replication. For simplicity, *T *will be used to refer to the unique unconstrained *T*_*j *_appearing in the analysis of the Choe *et al*. dataset.

### DEGs identification

After fitting the model on experimental observations two quantities are used to test if the probe-set corresponds to a DEG or not: *T*, or the *p*-value returned by testing if *T *is different from zero. In the results section, I will show that for the Choe *et al*. dataset there is a significant dependency between *T *and the average log_2 _probe intensity within a probe-set, *I*, that makes it unreasonable to use the default *T *= 0 as a null hypothesis for all probes.

To compensate this bias, the expected distribution of *T *and *I *over all probe-sets is estimated from the results returned by the linear model. A finite mixture of Gaussian components is known to be able to approximate any distribution and was used for this purpose. It is expected that one or several Gaussian component(s) will be affected to model probe-sets showing significant treatment effect and that this (those) component(s) should account for a proportion of the dataset corresponding to the expected fraction of DEGs. In typical microarray experiments, this fraction is usually assumed to be small (1–5% of the genes expected to change their level of expression). But in the validation dataset built by Choe *et al*. the fraction of DEG is known to be 9.3% (probe-sets with spiked ratios above 1). Moreover, the non-DEGs are known to fall in two distinct classes: 18.1% of cRNAs were amplified but kept in equal concentrations between the two samples and 72.4% of the cRNAs were not amplified. The segmentation of the probe-sets in three classes defined by the experimental setup warrants the use of a mixture of three Gaussians. And with this model, one Gaussian should be expected to model each class of probe-sets. DEGs can then be identified as the probe-sets with high conditional probabilities of belonging to the component of the mixture modeling DEGs, reducing this clustering step to an ordering of the probe-sets. In the case of real expression data, it is still unclear how to determine the optimal number of components to use and how to determine which component(s) are modeling the DEGs. Visual inspection of the data and mixture parameters are currently needed to make those decisions. A few observations are made in the discussion section regarding this aspect of the work.
